# Cryoglobulins as Potential Triggers of Inflammation in Schizophrenia

**DOI:** 10.1155/2013/125264

**Published:** 2013-12-15

**Authors:** Andranik Chavushyan, Meri Hovsepyan, Anna Boyajyan

**Affiliations:** Institute of Molecular Biology, National Academy of Sciences of the Republic of Armenia (NAS RA), 7 Hasratyan Street, 0014 Yerevan, Armenia

## Abstract

This case study aimed to investigate effects of type III cryoglobulins isolated from the blood of patients with schizophrenia on the production of proinflammatory cytokines interleukin(IL)-1**β**, IL-6 and tumor necrosis factor-**α** (TNF-**α**), anti-inflammatory cytokine IL-10, and chemotactic cytokines IL-8 and monocyte chemoattractant protein-1 (MCP-1) by peripheral blood mononuclear cells (PBMCs). The experiments were performed *in vitro* using PBMCs healthy subjects and the blood of patients whit schizoprenia. The enzyme-linked immunosorbent assay and 3-(4,5-dimethylthiazol-2-yl)-2,5-diphenyl tetrazolium bromide assay were used upon study. The results obtained indicated significant increase (*P* < 0.05) in IL-1**β**, IL-6, TNF-**α**, IL-8, and MCP-1 production by cultured PBMCs when incubating for 24 hours with cryoglobulins, beginning from 0.4 mg/mL. The gender difference does not affect the cryoglobulins-induced production of these cytokines by PBMCs. No influence of cryoglobulins on production of IL-10 by PBMCs was observed. Also, it was shown that cryoglobulins in concentration ≤4 mg/mL possessed no cytotoxic effect towards cultured PBMCs. Based upon the results obtained, we concluded that type III cryoglobulins are implicated in schizophrenia-associated alterations in the immune response through induction of the expression of proinflammatory and chemotactic cytokines by PBMCs.

## 1. Introduction

A considerable evidence suggests a role for upregulated immune response in the pathogenesis of schizophrenia (SCZ), since alterations in both the innate and adaptive immunity including autoimmune and inflammatory components were described in this pathology at both central and peripheral levels [1–7]. Moreover, according to a genetic-vascular-inflammatory hypothesis based upon a number of epidemiologic, clinical, and experimental studies, SCZ generates from a damage of the brain microvascular system initiated by genetically induced upregulated inflammatory reactions developed in response to ubiquitous environmental factors [8].

The results of our previous study revealed the detectable blood levels of type III cryoglobulins (Cgs) in SCZ and found the presence of complement activation split products in these complexes [9]. The presence of Cgs in the blood is detected in lymphoproliferative, autoimmune and infectious diseases and considered as a marker of the immune system chronic activation, inflammation, and autoimmune sensitization [10, 11]. Cgs can cause immune complex vacuities by depositing in the blood vessels, bind complement components, activate the complement system, and induce an inflammatory response [12–14]. Therefore, we proposed that in SCZ Cgs may be implicated in disease-associated inflammatory reactions.

On the basis of their immunoglobulin composition and total protein concentration Cgs were classified into three types. Type I (>5 mg/mL) consists of a monoclonal component alone, type II (>1 mg/mL) is a mixture of monoclonal and polyclonal immunoglobulins, and type III (<1 mg/mL) consists of a mixture of polyclonal immunoglobulins [15]. Recent finding suggested that type I Cgs (detected in lymphoproliferative disorders) stimulated secretion of tumor necrosis factor-*α* (TNF-*α*) by peripheral blood mononuclear cells (PBMCs) [16]. Data concerning the influence of type III and type II Cgs on cytokine production by PBMCs is missing.

The aim of this case study was to investigate the potential ability of type III Cgs isolated from the blood of SCZ patients to stimulate production by PBMCs of a number of cytokines including interleukin(IL)-1*β*, IL-6, TNF-*α*, IL-10, IL-8, and monocyte chemoattractant protein-1 (MCP-1). The choice of these mediators and modulators of the immune response was based upon the earlier reported data including also our own results indicating their altered levels in the blood of SCZ-affected subjects [5, 17–19].

## 2. Materials and Methods

### 2.1. Study Subjects

Fifty five patients with chronic paranoid SCZ (ICD-10 code: F20.0, DSM-IV-TR code: 295.30) treated with typical neuroleptic haloperidol (males/females: 34/21, mean age ± SD: 45.8 ± 8.4 years, mean age at the first-onset of illness ± SD: 17.4 ± 8.2 years, mean duration of illness ± SD: 28.4 ± 7.6 years), and 10 physically and mentally healthy subjects without family, past, or present history of SCZ or other psychiatric disorders (males/females: 5/5, mean age ± SD: 23.2 ± 1.2 years) were involved in this study. All patients were diagnosed by two independent experienced psychiatrists according to the presence of the relevant symptoms [20] and the results of the Structured Clinical Interview for DSM-IV (SCID) [21]. The affected subjects were recruited from the clinics of the Psychiatric Medical Center of the Ministry of Health of the Republic of Armenia (MH RA). The healthy subjects were recruited among the blood donors of the Erebouni Medical Center MH RA. Exclusion criteria for healthy subjects included psychiatric illness during lifetime, any serious neurological or endocrine disorder, any medical condition or treatment known to affect the brain, or meeting DSM-IV criteria for mental retardation as determined from the nonpatient version of the Structured Clinical Interview for DSM-IV-TR Axis I Disorders [22]. Exclusion criteria for all study participants included any serious medical disorder. All subjects gave their informed consents to participate in the study, which was further approved by the Ethical Committee of the Institute of Molecular Biology NAS RA (IRB #00004079).

### 2.2. Isolation of Cgs

Cgs were purified from the blood of affected subjects according to earlier described procedure [9] and kept at −30°C until further use. Before use, the Cgs samples were thawed, incubated at 37°C, and diluted with 0.15 M phosphate buffered saline (PBS) pH 7.4 (Oxoid Ltd.) to a required concentration. Concentration of Cgs was determined by measuring total protein according to the method of Lowry et al. [23] using bovine serum albumin as a standard.

### 2.3. Isolation and Cultivation of PBMCs: Study of Cgs-Induce Effects

PBMCs were obtained from heparinized blood of healthy subjects using standard Ficoll-Paque (Amersham Pharmacia Biotech) density gradient centrifugation. Thereafter PBMCs were diluted to 3 × 10^5^ cells/mL by Roswell Park Memorial Institute (RPMI)-1640 medium (Life Technologies) supplemented with 1% glutamine, 1% penicillin-streptomycin, 1% HEPES and 1% fetal bovine serum (complete culture medium) and incubated in the humidified incubator at 37°C with 5% CO_2_. The cell number was assessed using hemocytometer and optical microscope XDS-403 AT (Ningbo Wason Optical Instrument). In cytotoxicity test 3-(4,5-dimethylthiazol-2-yl)-2,5-diphenyl tetrazolium bromide (MTT) assay was used [24], and the relative number of cells was expressed as absorbance units at 550 nm (*A*_550_).

To determine whether Cgs from SCZ patients induce IL-10, IL-1*β*, IL-6, IL-8, TNF-*α*, and MCP-1 production by PBMCs, the latter were incubated with various concentrations of Cgs (range: 0.2–1.0 mg/mL of PBS) for 24 hours; in control samples only PBS was added. After incubation, the levels of cytokines were measured in culture medium. In order to assess the potential influence of gender difference on measured parameters, we compared the effects of Cgs from male patients with those from female patients on PBMCs from male and female healthy subjects, respectively.

### 2.4. IL-10, IL-1*β*, IL-6, IL-8, TNF-*α*, and MCP-1 Levels in Culture Medium

They were determined by enzyme-linked immunosorbent assay (ELISA) using commercially available kits (Gen-Probe Diaclone) according to manufacturer's instructions. In each assay we run duplicate of each sample, standard, and blank control (zero standard) on the same microplate. Experimental and control samples were run on the same microplate. Also, duplicates of the same samples (three of each) were run in each assay/on each microplate. The calculated overall intra-assay coefficient of variation (CV) was 5%, and the calculated overall interassay CV was 8%. Standard curves were reproducible with CV <4%. Concentration of cytokines was expressed as pg/mL.

### 2.5. Data Analysis

It was performed by GraphPad Prism-3.0 software (GraphPad Software Inc). Ordinal descriptive statistics, one-way ANOVA and Bonferroni multiple comparisons test were used for data analysis. Values of *P* < 0.05 were considered as significant. The data for each given parameter provided below in the text and figures represents the results of 10 independent experiments (with 3 repeated measurements in each) and is expressed as mean ± standard deviation (mean ± SD).

## 3. Results

**Figure 1:**
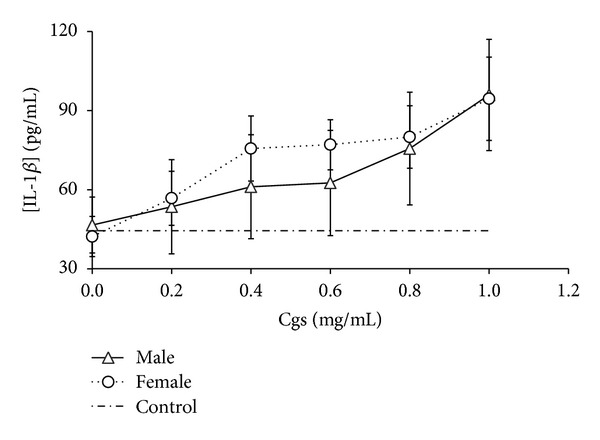
Increase in IL-1*β* concentration (pg/mL) in culture medium after the 24-hour incubation of PBMCs from male and female healthy subjects with Cgs isolated from the blood of male and female SCZ patients, respectively.

**Figure 2:**
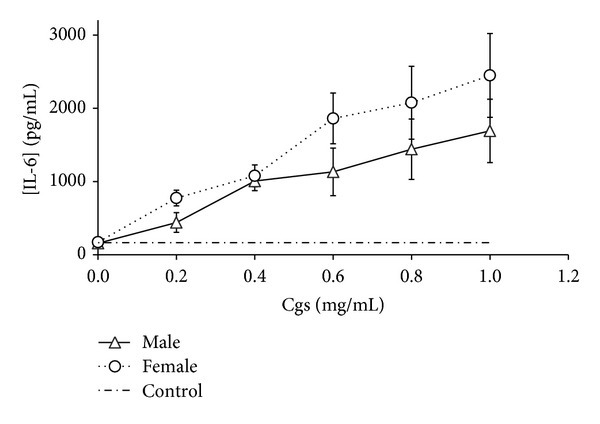
Increase in IL-6 concentration (pg/mL) in culture medium after the 24-hour incubation of PBMCs from male and female healthy subjects with Cgs isolated from the blood of male and female SCZ patients, respectively.

**Figure 3:**
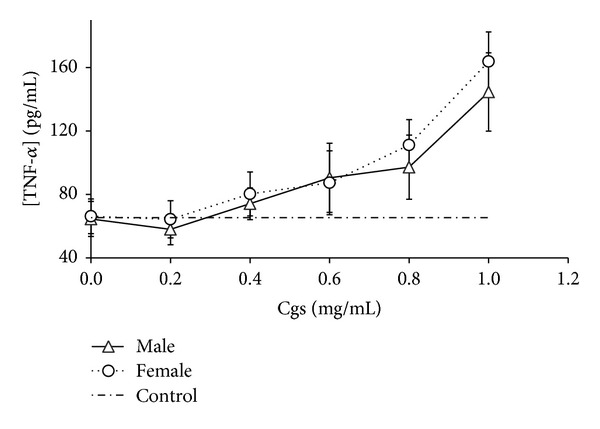
Increase in TNF-*α* concentration (pg/mL) in culture medium after the 24-hour incubation of PBMCs from male and female healthy subjects with Cgs isolated from the blood of male and female SCZ patients, respectively.

**Figure 4:**
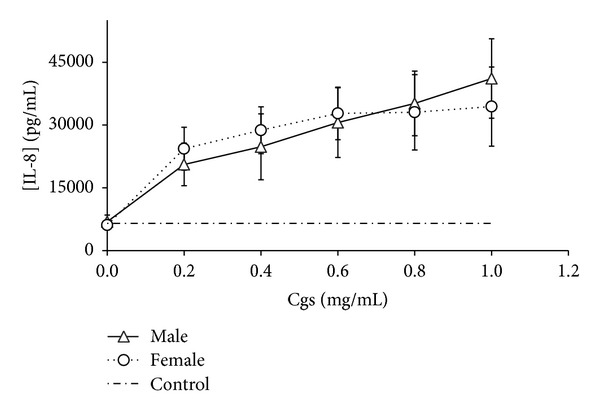
Increase in IL-8 concentration (pg/mL) in culture medium after the 24-hour incubation of PBMCs from male and female healthy subjects with Cgs isolated from the blood of male and female SCZ patients, respectively.

**Figure 5:**
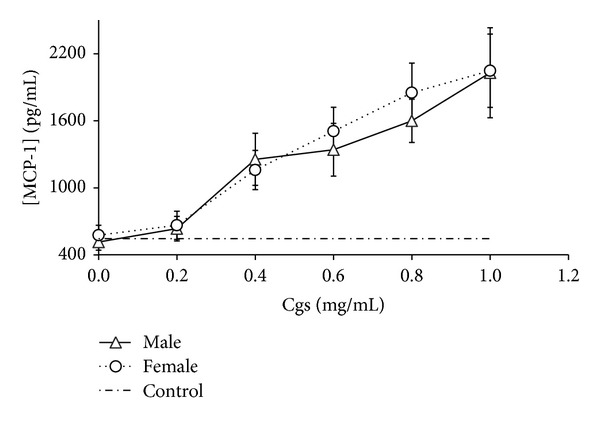
Increase in MCP-1 concentration (pg/mL) in culture medium after the 24-hour incubation of PBMCs from male and female healthy subjects with Cgs isolated from the blood of male and female SCZ patients, respectively.

We detected that Cgs (starting from concentration 0.4 mg/mL) induced production of proinflammatory and chemotactic cytokines IL-1*β*, IL-6, TNF-*α* and IL-8, MCP-1, respectively, by PBMCs (Figures 1, 2, 3, 4, and 5). Thus, after incubation of PBMCs with Cgs in concentration of 1 mg/mL, the mean levels of IL-1*β*, IL-6, TNF-*α*, IL-8, and MCP-1 in the culture medium were 2.1, 12, 2.4, 5.5, and 3.7 times significantly higher (*P* < 0.05) than basal levels of corresponding cytokines estimated before the incubation. While these effects were much more pronounced when Cgs and PBMCs from female subjects were used, the detected differences were statistically insignificant (*P* > 0.05) indicating that the gender difference does not affect the production of these cytokines by PBMCs induced by Cgs. In case of IL-10 no influence of Cgs on production of this anti-inflammatory cytokine by PBMCs was observed (data not shown).

**Figure 6:**
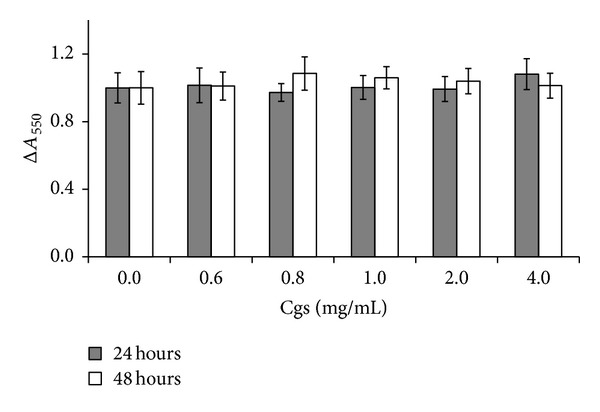
Relative number of PBMC (Δ*A*_550_) in the culture medium after incubation for 24 and 48 hours with indicated concentrations of Cgs isolated from the serum of patients with SCZ. Δ*A*_550_ = number of PBMC after incubation/number of PBMC before incubation. Δ*A*_550_ for each given data point is significant (*P* < 0.05).

To be sure that the detected effects are not conditioned by the cytotoxicity of Cgs, PBMCs were incubated with various concentrations of Cgs (range: 0.6–4.0 mg/mL) for 24 and 48 hours at 37°C. After incubation MTT assay was performed [24], and relative number of PBMCs before and after incubation was determined. According to the obtained results, Cgs isolated from SCZ patients in concentration ≤4 mg/mL had no cytotoxic effect on cultured PBMCs (Figure 6).

## 4. Discussion

The results obtained in the present study suggest that *in vitro* Type III Cgs isolated from the blood of SCZ patients may induce the expression of proinflammatory and chemotactic cytokines IL-1*β*, IL-6, TNF-*α* and IL-8, and MCP-1, respectively, by PBMCs. No influence of Cgs on anti-inflammatory cytokine IL-10 production by PBMCs was observed.

As it was already mentioned in the introduction earlier studies demonstrated increased levels of IL-1*β*, IL-6, TNF-*α*, IL-8, MCP-1 in the blood of SCZ-affected subjects [4–7, 17, 18, 25] providing evidence on the involvement of systemic inflammatory reactions in pathogenesis of SCZ. In addition, it was shown that monocytes of SCZ patients stimulated by lipopolysaccharide released significantly higher amounts of IL-1*β* and TNF-*α* than those of healthy subjects [26], and that leukocyte mRNA levels of TNF-*α* are significantly higher in first-episode SCZ patients then in healthy subjects [27]. Based upon the results obtained in the present study we concluded that Cgs may contribute to increased blood levels of these cytokines in SCZ and are involved in disease-associated activated peripheral inflammatory responses [1–3]. Our suggestion does not exclude the possibility that early reported genetic [5, 28, 29] or other environmental factors may be also responsible for altered blood levels of proinflammatory and chemotactic cytokines in SCZ.

Regarding IL-10, the increased blood levels of this cytokine earlier reported in patients with SCZ [13, 14] may be caused by early reported genetic factors [29–31] rather than by environmental.

Similar effects related to TNF-*α* and IL-10 were observed earlier upon studying the influence of type I Cgs on PBMCs. After the suppression of the complement activation, the reverse effect was detected, for example, decrease in TNF-*α* production and increase in IL-10 production by PBMCs in the presence of type I Cgs [16]. While IL-10 is known as inhibitor of TNF-*α* expression [32], it seems that in SCZ this regulatory mechanism does not work, since the increased levels of both cytokines were detected in this pathology [5, 18, 19].

Cgs can induce production of proinflammatory cytokines by PBMCs via Fc receptors as it was demonstrated in case of circulating immune complexes for TNF-*α* [33]. In addition, our previous study revealed the presence of the C1q complement protein and C3-derived opsonins, natural ligands of CR1 complement receptor, in Cgs isolated from the blood of SCZ patients [9]. Binding of C3b-containing Cgs to CR1 on monocytes will induce IL-1*β* release, and binding of C1q-containing Cgs to CR1 on PBMCs will induce the activation of the complement as it usually occurs in case of circulating immune complexes [34, 35]. Since the activation of the complement stimulates the expression of chemotactic cytokines [36], we propose that the effects of Cgs towards IL-8 and MCP-1 production by PBMCs may be realized by a complement dependent mechanism. Hyperactivation of the complement cascade in SCZ was demonstrated in a number of studies [37], thus this complement-dependent mechanism may be implicated in Cgs-induced effects both *in vivo* and *in vitro*. However, direct induction of chemokine expression by Cgs via C1qR receptors, as it was described for IL-8 in case of C1q-containing circulating immune complexes [38], cannot be excluded. Further investigations will help to clear these issues.

Limitation of this study is the involvement of patients treated with antipsychotic (typical neuroleptic haloperidol). However, it has to be mentioned that our previous study reveals no different in Cgs blood levels and composition between neuroleptic-treated and nontreated patients with schizophrenia [9].

## 5. Conclusions

In summary, we concluded that type III Cgs are implicated in SCZ-associated alterations in the immune response through induction of the expression of proinflammatory and chemotactic cytokines by PBMCs.
